# Vacancy‐Contained Tetragonal Na_3_SbS_4_ Superionic Conductor

**DOI:** 10.1002/advs.201600089

**Published:** 2016-04-23

**Authors:** Long Zhang, Dechao Zhang, Kun Yang, Xinlin Yan, Limin Wang, Jianli Mi, Bo Xu, Yueming Li

**Affiliations:** ^1^ State Key Laboratory of Metastable Materials Science and TechnologyYanshan UniversityQinhuangdaoHebei066004P.R. China; ^2^ Institute of Solid State PhysicsVienna University of TechnologyWiedner Hauptstr8‐10, 1040ViennaAustria; ^3^ Institute for Advanced MaterialsSchool of Materials Science and EngineeringJiangsu UniversityZhenjiangJiangsu212013P.R. China

**Keywords:** chalcogenide, sodium batteries, sodium superionic conductors, solid electrolytes, sulfides

## Abstract

**Tetragonal Na_3_SbS_4_** is synthesized as a new sodium superionic conductor. The discovery of Na vacancies experimentally verifies previous theoretical predictions. Na vacancies, distorted cubic sulphur sublattices and large Na atomic displacement parameters lead to the ionic conductivity as high as 3 mS cm^−1^, a value significantly higher than those of state‐of‐the‐art sodium sulfide electrolytes.

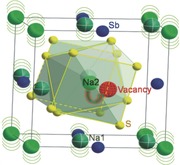

The growing demand for high capacity rechargeable batteries used in large‐scale energy storage,^1^ including electric vehicles and electrical grid energy storage, has promoted the development of sodium all‐solid‐state batteries.^2^ As a type of solid electrolyte (SE), sulfide superionic conductors have been attracted worldwide attention because of its relatively high room temperature ionic conductivity as well as low grain‐boundary resistance achieved by directly cold‐pressing the sulfide powders, which makes them favorable to assemble all‐solid‐state batteries.^3^ Significant progress has been made in lithium sulfide electrolytes in recent years.[[qv: 2f,3,4]] However, in spite of the similar characteristic to lithium, the milestone of sulfide sodium superionic conductors as a room‐temperature SE was ambiguous until Hayashi and co‐workers introduced cubic Na_3_PS_4_ with ionic conductivity of 0.2 mS cm^−1^ in 2012,^5^ and subsequently 0.46^6^ and 0.74^7^ mS cm^−1^ by using high purity starting materials and Si doping, respectively. These values are two orders of magnitude higher than that of tetragonal Na_3_PS_4_.^8^ After that, impressive breakthroughs on both theoretical and experimental researches have been achieved for sulfide‐based sodium SEs.^9^ Nonetheless, there are much fewer sodium superionic conductors in contrast to lithium sulfides; their room‐temperature ionic conductivities remain low. Exploration of sodium sulfides with ionic conductivity over 1 mS cm^−1^ is highly anticipated.

Ceder and co‐worker proposed design principles for superionic conductors and suggested that the anion sulphur sublattices analogous to body‐centered cubic (bcc) frameworks allow the migration of ions with a lower activation barrier than in other close‐packed frameworks, thus resulting in fast ion diffusion.^10^ Moreover, cell volume has been reported to play an important role in either Na^+^ or Li^+^ transport, such as the latest works on Se‐[[qv: 9f]] and Sn‐doped[[qv: 9e]] Na_3_PS_4_ (with expanded unit cell) as well as cation‐substituted Li_10_GeP_2_S_12_ (LGPS).^11^ Very recently, theoretical investigations on both Na_3_PS_4_[[qv: 9e]] and Na_3_PSe_4_[[qv: 9g]] revealed that a defect‐driven diffusion mechanism (either Na^+^ interstitial or Na^+^ vacancy) accounts for the high ionic conductivity, while the stoichiometric compounds showed negligible diffusivity. Actually, Na^+^ deficiency is the reality during synthesis as it is a very reactive metal. Therefore, an ultrafast ion diffusion is expected if the conductor meets the prerequisites of large cell volume, low migration barrier energy, and/or sodium vacancy defects.

In this study, Na_3_SbS_4_ with a tetragonal structure was synthesized and evaluated for the first time. An expanded unit cell containing distorted‐cubic sulphur sublattices was identified. Moreover, the existence of Na vacancies in Na_3_SbS_4_ structure was revealed, experimentally verifying the vacancy theory recently proposed.[[qv: 9g]] This tetragonal Na_3_SbS_4_ demonstrates a high ionic conductivity of 3 mS cm^−1^ and is the fastest room temperature Na^+^ solid conductor to date.

The Rietveld refinement results for X‐ray diffraction (XRD) pattern of synthesized Na_3_SbS_4_ is shown in **Figure** **1**a. The halo patterns at low angle reflect the polyimide film. The crystal structure was determined to be tetragonal with the space group $P\bar{4}2_{1}c$ (No. 114) with two formula units per unit cell. To the best of our knowledge, this tetragonal Na_3_SbS_4_ has not been reported yet. The plots (Figure 1a) of the observed, calculated, and difference patterns from the Rietveld refinement prove undoubtedly the formation of single‐phase tetragonal Na_3_SbS_4_. **Table** **1** lists the refined structural data and refinement parameters. The cell parameters of Na_3_SbS_4_ are *a*,*b* = 7.1597 (5) Å and *c* = 7.2906(6) Å, larger than those of tetragonal Na_3_PS_4_ (*a,b* = 6.9520 (4) Å, *c* = 7.0757(5) Å)^8^ and Si‐doped cubic Na_3_PS_4_ (*a* = 6.9978 Å),^12^ while lower than that of cubic Na_3_PSe_4_ (*a* = 7.3094(2) Å).[[qv: 9f]] Tetragonal Na_3_SbS_4_ is just slightly deviated from its cubic symmetry^13^ by elongating *c* axis.

**Figure 1:**
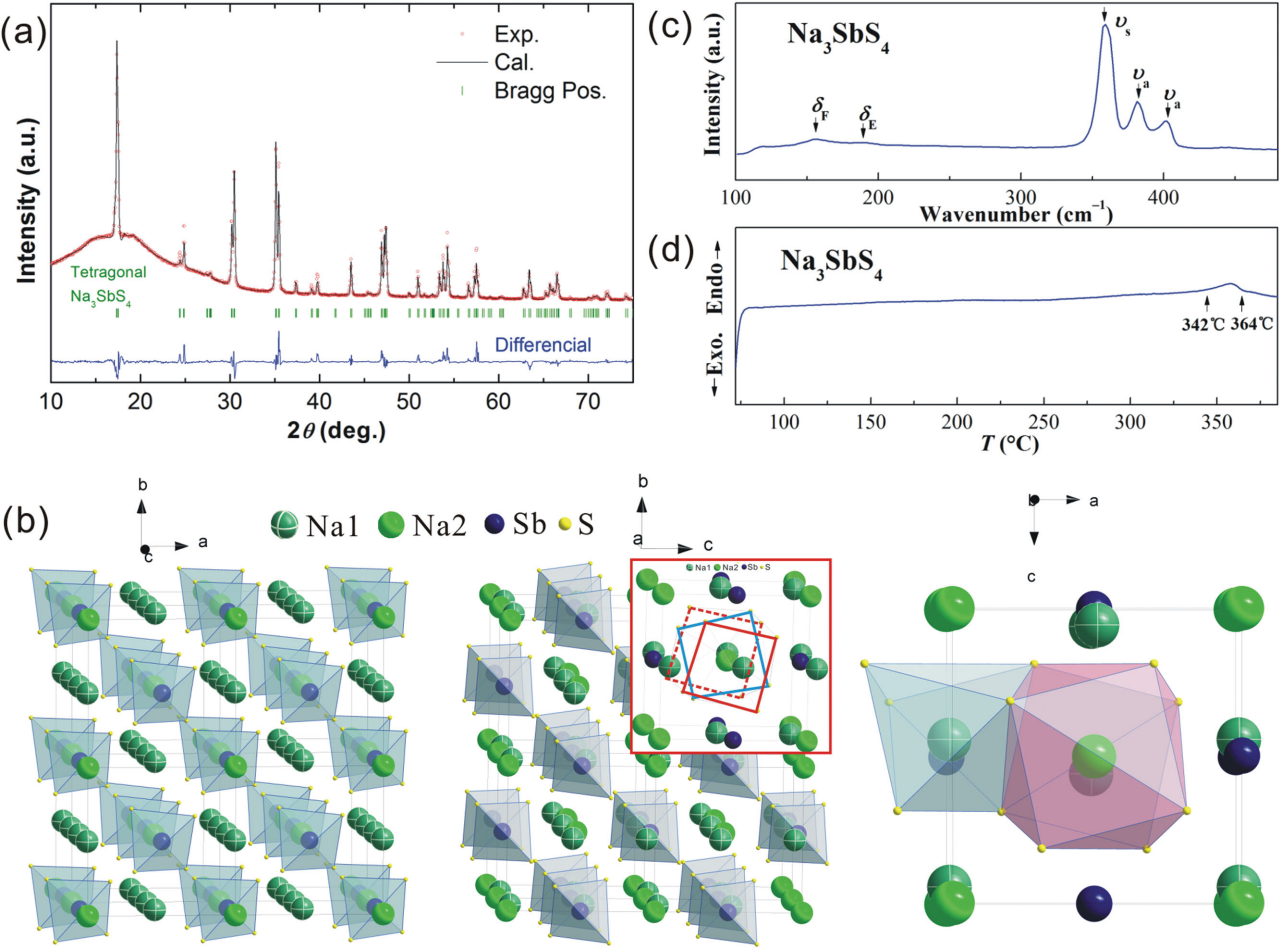
a) Rietveld refinement of the powder diffraction pattern for tetragonal Na_3_SbS_4_. b) The crystal structure of Na_3_SbS_4_ viewed along *c* (left pannel) and *a* axes (middle pannel), and Na1 and Na2 coordination environments (right pannel). The inset of middle panel illustrates diffusion channels along *a*‐direction composed by S‐anion distorted cubic sublattices. c) Raman spectrum of Na_3_SbS_4_ at ambient condition. d) Differential scanning calorimetry curve of Na_3_SbS_4_.

**Table 1:** X‐ray powder diffraction data (room temperature) for Na_3_SbS_4_ from Rietveld refinement; *B*_iso_ values in (10^2^ nm^2^)

Compound	Na_3_SbS_4_
Refined composition (at.%)	Na_2.8_SbS_4_□_0.2_
*a = b*, *c* (Å)	7.1597(5), 7.2906(6)
*Z* ^a)^	2
Number of variables	51
*R_F_* = Σ|*F_o_* – *F_c_*|/Σ*F_o_*	0.064
*R_I_* = Σ|*I_o_* – *I_c_*|/Σ*I_o_*	0.084
*R_wP_* = [Σ*w_i_*|*y_oi_* – *y_ci_*|^2^/Σ*w_i_*|*y_oi_*|^2^]^1/2^	0.078
*R_P_* = Σ|*y_oi_ – y_ci_*|/Σ|*y_oi_*|	0.049
*R_e_* = [(*N – P + C*)/(Σ*w_i_y* ^2^ *_oi_*)]^1/2^	0.015
Sb, in 2*b* (0, 0, ½), *B*_iso_	0.40(9)
Na(1), in 4*d* (0, ½, *z*), *z*, *B*_iso_	0.4412(16), 1.7(3)
Na(2), in 2*a* (0, 0, 0), Occ., *B*_iso_	1.6(1)Na+0.4□, 1.9(5)
S, in 8*e* (*x*, *y*, *z*), *x*, *y*, *z*, *B*_iso_	0.2917(9), 0.3307(8), 0.1884(4), 0.9(2)

^a)^ *Z*: number of formula units per unit cell.

Surprisingly, unlike other sodium sulfides showing stoichiometric composition, Rietveld refinement revealed the vacancy existence at Na2 sites (see Table 1), experimentally verifying the defect hypothesis previously proposed.[[qv: 9e–g]] The occupancy of Na2 sites contains 80% Na plus 20% vacancies rather than full occupancy of Na. The mole fraction of vacancies in Na_3_SbS_4_ is calculated to be 2.5%, comparable to the hypothesis value of 2.1% for Na_3_PSe_4_ assumed by Bo et al.[[qv: 9g]] With theoretical calculation,[[qv: 9g]] Ceder and co‐workers found that vacancy defect has the lowest formation energy among different defects evaluated and is the key factor for accelerating Na^+^ diffusion in Na_3_PSe_4_. A extrapolated ionic conductivity as high as 28.9 mS cm^−1^ at room temperature for Na_3_PSe_4_ with 2.1 mol.% Na vacancies was reported.[[qv: 9g]]

The crystal structures of tetragonal Na_3_SbS_4_ along various viewing directions are given in Figure 1b. The crystallographic unit cell consists of two SbS_4_ tetrahedral groups, with Sb atoms sit on 2*b* sites and S atoms on 8*e* sites. The Sb–S distance is 2.3619 Å and the S–Sb–S angles are 109.77° and 108.88°, indicating a slightly distorted regular tetrahedron and deviation of Sb atoms from the center of the tetrahedron. In contrast to cubic Na_3_SbS_4_ where Na atoms situate simply at 6*b* site,^13^ tetragonal Na_3_SbS_4_ shows two independent Na positions: Na1 at 4*d* and Na2 at 2*a* sites (Table 1). Linear (Na1) or zigzag (Na1 and Na2) rows of Na^+^ occupy the interstices composed of the SbS_4_ tetrahedrons. The former locates in the channels parallel to the crystallographic *c* axis (left panel to Figure 1b) while the latter in the channels parallel to *a* or *b* axis (middle panel to Figure 1b). All these Na^+^ diffusion channels construct along 3D pathways orthogonal to each other. It is notable that the sulphur sublattices (inset of middle panel to Figure 1b) demonstrate an intensely distorted cubic lattice. This anion framework may benefit Na^+^ diffusion followed the mechanism similar to the bcc‐sublattice‐contained superionic conductors described in previous study.^10^ Different coordinations are observed for these two types of Na^+^, i.e., Na at Na1 and Na2 sites bound to six and eight sulphur atoms, respectively, as shown in the right panel to Figure 1b. The Na1–S distances are 2.80, 3.07, and 3.13 Å and the Na2–S distances are 3.02 and 3.38 Å.

Considering the existence of vacancies, the expanded unit cell, and the cubic‐like sulphur sublattices, it is not surprising that the isotropic atomic displacement parameter (*U*_iso_ = *B*_iso_/8*π* ^2^) of Na at either Na1 (0.022(4) Å^2^) or Na2 (0.024(6) Å^2^) sites is significantly larger than those for Sb (0.005(1) Å^2^) and S (0.011(3) Å^2^), as listed in Table 1. Fast Na^+^ diffusion is consequently highly expected.

Figure 1c shows the Raman spectrum of Na_3_SbS_4_ at room temperature. The prominent resonance peaks observed over the measured range are attributed to the Sb–S vibrations of the isolated SbS_4_ group and obviously different from those of SbS_3_ group,^14^ confirming the successful formation of SbS_4_ group. The peaks at 402 (*ν*_a_), 382 (*ν*_a_), and 359 (*ν*_s_) cm^−1^ are assigned to the stretching vibration modes and those at 189 (*δ*_E_) and 155 (*δ*_F_) are assigned to the deformational vibration modes of Sb–S(4).[[qv: 14a]] Neither phase transition nor decomposition is observed from differential scanning calorimetry (DSC) profile until 342 °C with a small endothermic peak (Figure 1d), confirming the thermal stability of tetragonal Na_3_SbS_4_. The endothermic reaction may be related to a decomposition of impurity phase that is out of the resolution of XRD.

Scanning electron microscopy (SEM) images for the morphology of Na_3_SbS_4_ pellets from hand‐ground and ball‐milled powders are displayed in **Figure** **2**. The cold‐pressed pellet from hand‐ground powders shows more or less isolated grains with irregular shapes (Figure 2a), whereas that from ball‐milled powders shows grains with more smooth shape and better connections (Figure 2b). However, the contacts between grains of the latter are inferior to those of ball‐milled Na_3_PS_4_ reported previously.^5^ Further optimization on preparation process to improve the pellet's density is ongoing.

**Figure 2:**
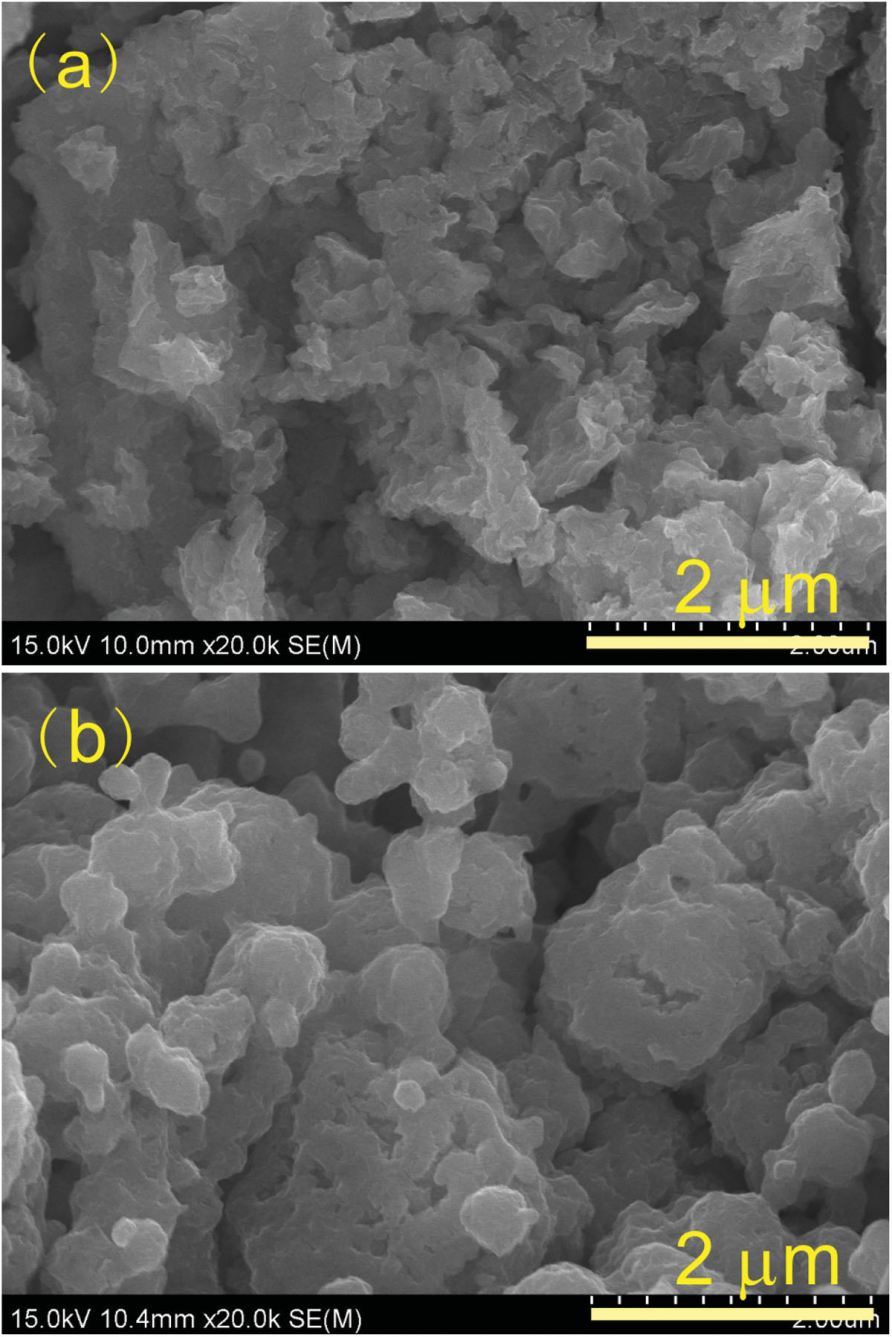
SEM fracture images of Na_3_SbS_4_ cold‐pressed from a) the melted ingot pulverized by hand grinding in an agate mortar and b) ball‐milled powders.

Arrhenius plot of the total ionic conductivity *σ* in the temperature range from 25 to 90 °C is depicted in **Figure** **3**a for Na_3_SbS_4_. Nyquist plots of the impedance at different temperature are shown in the inset. The total ionic conductivity is calculated from the local minimal resistance at the intersection of the impedance spectrum. The relation log*σ*∼1/T obeys the Arrhenius law, confirming a high purity and stable phase of the Na_3_SbS_4_ compound. The ionic conductivity locates in the range from 3 mS cm^−1^ at 25 °C to 16 mS cm^−1^ at 90 °C, approaching to those of organic liquid electrolytes currently used.[[qv: 2a]] This value is three orders of magnitude larger than that of tetragonal Na_3_PS_4_ 8 and significantly higher than those of state‐of‐the‐art sulfides with cubic structure.[[qv: 9f,g]] The activation energy *E*_a_ for the sodium‐ion conduction were determined from the slope of the linear Arrhenius plot using the equation:^15^ *σ* = *σ*_o_ exp(–*E*_a_/*k*_B_ *T*), where *σ*_o_ represents the pre‐exponential parameter and *k*_B_ the Boltzmann constant. The calculated activation energy of Na_3_SbS_4_ is 0.25 eV, comparable to those of LGPS and Na_3_PS_4_ sulfides.^5^, ^16^ Na_3_SbS_4_ with the high ionic conductivity and low activation energy can be considered as a promising SE used in all‐solid‐state sodium ion batteries.

**Figure 3:**
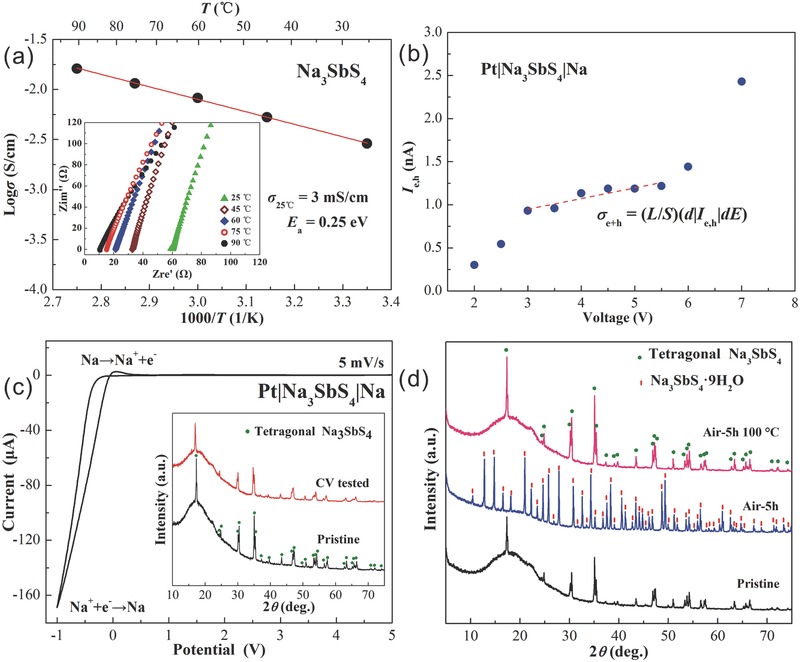
a) Arrhenius conductivity plot of tetragonal Na_3_SbS_4_ from 25 to 90 °C. The inset displays the impedance spectra of Na_3_SbS_4_ measured at different temperature. b) Wagner's polarization curve measured on a Pt/Na_3_SbS_4_/Na cell at room temperature. c) Cyclic voltammogram of Na_3_SbS_4_. The inset displays XRD profiles of Na_3_SbS_4_ before and after CV measurements. d) XRD patterns of Na_3_SbS_4_ powders before and after air‐exposure for 5 h, and heated at 100 °C after air‐exposure.

The impedance spectrum of a solid ion conductor generally contains three distinct features, i.e., two semicircles at high and intermediate frequency and a Warburg‐type region at low frequency, which are assigned to intragrain transport (bulk), intergrain transport (grain boundary), and capacitive behavior, respectively. In case of Na_3_SbS_4_, the semicircles (inset of Figure 3a) are hardly to be observed, especially measured at high temperature range, indicating a negligible intergrain transport. The inherently low grain boundary resistance is a typical characteristic of sulfides, which have the advantage to be assembled without heat treatment.^3^, ^17^ The steep linear spike at low frequency indicates that Na_3_SbS_4_ is a typical ionic conductor.^18^

The total conductivity generally includes contributions from different charge carrying species. A SE normally involves two kinds of conductive species including: dominant ions (*σ*_Na_^+^ for sodium ionic conductors) and minor electrons and/or holes (*σ*_(_ *_e_*_+_ *_h_*_)_). The total conductivity of Na_3_SbS_4_ can be expressed by *σ* = *σ*_Na_^+^ + *σ*_(_ *_e_*_+_ *_h_*_)_. The transference number for sodium ions is accordingly derived by *t*_Na_^+^ = *σ*_Na_^+^/*σ*. Wagner's polarization measurements were performed at room temperature to determine *σ*_(_ *_e_*_+_ *_h_*_)_. The resulting current–voltage curve is illustrated in Figure 3b. The total electronic contribution *σ*_(_ *_e_*_+_ *_h_*_)_ at the irreversible Na_3_SbS_4_/Pt side can be calculated by linear fitting the current–voltage curve in the voltage range from 3 to 5.5 V using the equation:^19^ *σ*_(_ *_e_*_+_ *_h_*_)_. = (*L*/*S*)(*d*|*I_e_*_,_ *_h_*|/*dE*), where |*I*_e,h_| is the total electronic current, *E* is the polarization voltage, *S* is the electrolyte–electrode surface area, and *L* is the electrolyte thickness. The calculated value is *σ*_(_ *_e_*_+_ *_h_*_)_. = 2 × 10^−8^ mS cm^−1^, eight orders of magnitude lower than the total ac conductivity (3 mS cm^−1^). It thus can be concluded that *t*_Na_^+^ practically reaches unity and ion can be regarded as the sole conductive species.

The electrochemical stability of Na_3_SbS_4_ with metallic sodium was evaluated using cyclic voltammetry (CV) measurement on a Pt/Na_3_SbS_4_/Na cell, as shown in Figure 3c. The potentials were scanned from −1 to 5 V versus Na/Na^+^ at a scan rate of 5 mV s^−1^. The SE shows a broad electrochemical window up to 5 V, other than the sodium deposition and dissolution because of the cathodic and anodic reactions near 0 V versus Na/Na^+^, indicating a high decomposition potential. However, the low anodic peak suggests the deposited sodium is only partially stripped during the anodic reaction while the remains reacted with the electrolyte to produce an inactive corrosion product.^20^ XRD patterns of Na_3_SbS_4_ before and after three circles CV measurements are displayed in the inset of Figure 3c. No extra peak is observed after CV tests, suggesting a relative stability of Na_3_SbS_4_ against Na metal, e.g., Na_3_SbS_4_ is more stable against Na metal than Na_3_PSe_4_,[[qv: 9f]] whereas less stable than Na_3_PS_4_.^5^ Similar to the formation of Li_2_S in lithium‐sulfides against Li metal reported previously,^21^ small amount of Na_2_S may form during the reduction process and act as a passivation layer mitigating the decomposition of Na_3_SbS_4_. The instability of Na_3_SbS_4_ toward metallic Na is a drawback of sulfide SEs.^21^ This issue could be solved through doping,^22^ surface decoration,^23^ and forming passivation layers.^24^ Our future work will concentrate on improving the stability of Na_3_SbS_4_ against Na metal and assembling all‐solid‐state batteries using the Na_3_SbS_4_ solid electrolyte to investigate the electrochemical performance.

The hygroscopic characteristic of Na_3_SbS_4_ was investigated in air with 65% humidity. Figure 3d shows the XRD patterns for Na_3_SbS_4_ samples before (pristine) and after air‐exposure for 5 h (air‐5h) and followed by heating at 100 °C (air‐5h 100 °C). Na_3_SbS_4_ absorbs the moisture in air and forms Na_3_SbS_4_·9H_2_O (PDF#43‐0442). Na_3_SbS_4_ is moisture sensitive similar to many other sulphides. Enhancement of air stability is feasible by appropriate materials′ design, as described in previously studies.^15^, ^25^ However, single phase tetragonal Na_3_SbS_4_ is fully recovered from dehydration of Na_3_SbS_4_·9H_2_O at 100 °C, indicating a completely reversible hydration and dehydration process for Na_3_SbS_4_, which is favorable for Na_3_SbS_4_ preparation from solution as well as storage in air.

In summary, a new sodium superionic conductor tetragonal Na_3_SbS_4_ with the space group $P\bar{4}2_{1}c$ was synthesized and investigated for the first time. Na atoms at both 2*a* and 4*d* sites demonstrate large isotropic atomic displacement parameters. Sulphur sublattices were found to be an intensely distorted cubic lattice. Most notably, the existence of 2.5 mol. % Na vacancies at Na2 sites (2*a*) was discovered and experimentally verifies previous hypothesis. Benefit from these favorable features, tetragonal Na_3_SbS_4_ shows prominent performance with fast ion diffusion. With a Na^+^ transference number approaching unity, the ionic conductivity reaches 3 mS cm^−1^ at room temperature, the best value among state‐of‐the‐art sodium sulfides to date to the best of our knowledge. Na_3_SbS_4_ is a promising candidate for practical application as an SE in all‐solid‐state sodium ion batteries.

## Experimental Section

*Synthesis of Na_3_SbS_4_*: Na metal (AR, Sinopharm), Sb (99.999%, Sinopharm), and S (99.999%, Alfa) powder were mixed according to stoichiometric proportion of Na_3_SbS_4_. for sequential solid‐state reaction. The mixture was loaded into a glassy carbon crucible, which was vacuum‐sealed in a quartz tube, slowly heated to 700 °C in order to avoid extensive exothermal reaction, dwelled for 12 h and cooled down naturally in the furnace. Based on our experiences, powders with coarse particles are not favorable for cold pressing. The resultant ingot was thus ball‐milled by using a Fritsch planetary mill with 10 mm diameter balls. All procedures were carried out in an argon‐filled glove box with H_2_O, O_2_ < 0.5 ppm.

*Materials Characterization*: XRD was performed using a Rigaku D/MAX‐2500/PC (Cu K*α*, 40 kV 200 mA). The powders were sealed in an air‐tight container and covered with polyimide film to prevent moisture. The crystal structure was solved by using the direct space method and was then refined by the Rietveld method. Raman scattering measurements were performed using a Renishaw inVia system with a 514.5 nm excitation source. DSC profiles were recorded on a Perkin‐Elmer DSC8000 with a scan rate of 10 °C min^−1^. Fracture images were taken with a Hitachi S‐4800 II FESEM.

*Electrochemical Tests*: Electrochemical Impedance Spectroscopy measurements were performed in the frequency range of 0.1 Hz to 2 MHz at room temperature using a Princeton P4000 impedance analyzer. The pellets for measurements were cold‐pressed from ball‐milled powders at 400 MPa. Indium foil was placed on both sides of the pellets as electrodes. Stainless‐steel rods were then attached to both sides as current collectors in an air‐tight two‐electrode cell. Wagner's polarization measurements were performed at room temperature to determine the electronic contribution to the total conductivity on a Pt/Na_3_SbS_4_/Na cell. The cell was polarized by applying a constant dc potential (Princeton P4000) from 2 to 7 V across the Pt blocking electrode and the Na reversible electrode. The voltage was held at each step for 1000 s, approaching to a stationary state in each step of chronoamperometric experiment. The CV measurements were carried out at voltages ranging from −1 to 5 V with a scan rate of 5 mV s^−1^ in an asymmetric Pt/Na_3_SbS_4_/Na cell, where Pt is a working electrode and Na a counter/reference electrode. A Faraday cage was used for all electrochemical measurements.

[Further details of the crystal structure investigation may be obtained from the Fachinformationszentrum Karlsruhe, 76344 Eggenstein‐Leopoldshafen (Germany), on quoting the depository number CSD‐430596.]
